# Climate change induces multiple risks to boreal forests and forestry in Finland: A literature review

**DOI:** 10.1111/gcb.15183

**Published:** 2020-06-13

**Authors:** Ari Venäläinen, Ilari Lehtonen, Mikko Laapas, Kimmo Ruosteenoja, Olli‐Pekka Tikkanen, Heli Viiri, Veli‐Pekka Ikonen, Heli Peltola

**Affiliations:** ^1^ Finnish Meteorological Institute Helsinki Finland; ^2^ School of Forest Sciences University of Eastern Finland Joensuu Finland; ^3^ UPM Forest Tampere Finland

**Keywords:** bark beetles, boreal forests, climate change, drought, forest damage, forest fire, heavy snow loading, *Heterobasidion* root rot, wind damage

## Abstract

Climate change induces multiple abiotic and biotic risks to forests and forestry. Risks in different spatial and temporal scales must be considered to ensure preconditions for sustainable multifunctional management of forests for different ecosystem services. For this purpose, the present review article summarizes the most recent findings on major abiotic and biotic risks to boreal forests in Finland under the current and changing climate, with the focus on windstorms, heavy snow loading, drought and forest fires and major insect pests and pathogens of trees. In general, the forest growth is projected to increase mainly in northern Finland. In the south, the growing conditions may become suboptimal, particularly for Norway spruce. Although the wind climate does not change remarkably, wind damage risk will increase especially in the south, because of the shortening of the soil frost period. The risk of snow damage is anticipated to increase in the north and decrease in the south. Increasing drought in summer will boost the risk of large‐scale forest fires. Also, the warmer climate increases the risk of bark beetle outbreaks and the wood decay by Heterobasidion root rot in coniferous forests. The probability of detrimental cascading events, such as those caused by a large‐scale wind damage followed by a widespread bark beetle outbreak, will increase remarkably in the future. Therefore, the simultaneous consideration of the biotic and abiotic risks is essential.

## Graphical abstract

In Finland, projected climate change acts to increase forest growth by the mid‐21st century, particularly in the northern parts of the country. Nonetheless, in the south, the growing conditions may become suboptimal for Norway spruce due to drought and excessively high temperatures. Biotic damages, mainly caused by Spruce bark beetles and Heterobasidions, are anticipated to increase in southern Finland. Abiotic disturbances due to windstorms, drought and forest fires will be exacerbated especially in the south with snow damages in the north. Climate change likewise increases detrimental cascading events, for example, large‐scale wind damages followed by a widespread bark beetle outbreak.

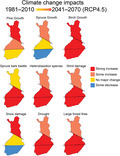

## 1 INTRODUCTION

The magnitude of future emissions of the greenhouse gases (GHGs) and the development of their atmospheric concentrations affect the energy balance of the Earth and global climate. Several alternative GHG scenarios, termed Representative Concentration Pathways (RCPs), have been developed (van Vuuren et al., 2011). Under the most optimistic RCP2.6 scenario, climate change mitigation succeeds well, whereas under the most pessimistic RCP8.5 scenario mitigation fails totally. Under the RCP2.6, RCP4.5 and RCP8.5 scenarios, the global mean temperature will rise about 1, 2 and 4°C by 2100, respectively, compared with the recent period of 1986–2005 (IPCC, 2013). Due to the feedback mechanisms in the climate system, the warming will be larger at high latitudes in northern Europe (i.e. boreal zone) than close to the equator. It is also larger in winter than in summer. The precipitation is also projected to increase in the boreal zone, especially during cold seasons (Ruosteenoja, Jylhä, & Kämäräinen, 2016).

According to previous studies, climate change may have both positive and negative impacts on boreal forests and forestry (e.g. Kellomäki, Peltola, Nuutinen, Korhonen, & Strandman, 2008; Kellomäki et al., 2018; Reyer et al., 2014, 2017; Subramanian, Bergh, Johansson, Nilsson, & Sallnäs, 2016). Overall, forest growth is likely to increase in Nordic countries (e.g. Kellomäki et al., 2008, 2018; Poudel et al., 2011, 2012) where a relatively short growing season, low summer temperatures and supply of nutrients currently limit the growth (e.g. Hyvönen et al., 2007; Kellomäki et al., 2008). Additionally, the elevation of the atmospheric CO_2_ concentration acts to enhance forest growth further, due to an increase in the water use efficiency (see e.g. Ellsworth et al., 2012; Hyvönen et al., 2007; Kellomäki et al., 2018; Peltola, Kilpeläinen, & Kellomäki, 2002). This is related to a simultaneous decrease in transpiration and an increase in the photosynthetic rate under elevated CO_2_ (Medlyn et al., 1999, 2001).

On the other hand, future forest growth (and productivity) is likewise affected by current forest structure, the intensity of forest management and harvesting (Garcia‐Gonzalo, Peltola, Zubizarreta Gerendiain, & Kellomäki, 2007; Heinonen et al., 2018; Poudel et al., 2012). Strongly increasing temperatures and drought episodes may also make the growing conditions suboptimal for some tree species, like Norway spruce (*Picea abies*), especially in the southern boreal zone (see e.g. Allen et al., 2010; Kellomäki et al., 2008, 2018).

Climate change is likely to increase many abiotic and biotic damages in European forests, such as those caused by windstorms, heavy snow loading, drought, forest fires and major insect pests and pathogens of trees (Bentz et al., 2019; Jactel et al., 2011; Reyer et al., 2017; Seidl, Schelhaas, Rammer, & Verkerk, 2014; Seidl et al., 2017). During the past few decades, windstorms have damaged a significant amount of timber and caused substantial economic losses for forestry in central and northern Europe (Gregow, Laaksonen, & Alper, 2017; Reyer et al., 2017; Schelhaas, Nabuurs, & Schuck, 2003; Seidl et al., 2014). The increasing amount of wind damage in past decades can, at least partly, be explained by an increasing volume of growing stock and changes in forest structure (Schelhaas et al., 2003; Seidl, Schelhaas, & Lexer, 2011).

At the all‐European level, snow‐induced damages have typically been minor compared to the damage caused by windstorms (Schelhaas et al., 2003). Snow damages have been most frequent in northern Europe and at high altitudes (Nykänen, Broadgate, Kellomäki, Peltola, & Quine, 1997). However, under the warming climate both the risks of wind and snow‐induced damage may increase, due to the decreased duration of frozen soil (Kellomäki, Maajärvi, Strandman, Kilpeläinen, & Peltola, 2010; Lehtonen et al., 2019; Venäläinen et al., 2001), which weakens the anchorage of trees during the windiest season from late autumn to early spring. Warming climate and an increase in drought events may also increase the frequency of forest fires, which has already been observed particularly in south‐eastern Europe (Venäläinen et al., 2014). The widespread and devastating fires in Sweden in 2014 and 2018 demonstrated that such calamities are possible also in the cool climate of the Nordic countries.

Warmer climate also favours many current and potential new insect pests and pathogens of trees, both directly (e.g. through the expansion of living area and abundance of pests) and indirectly (e.g. as a consequence of physical damage caused by storm and forest fires). The most significant insect pest in the European forests is the European spruce bark beetle, *Ips typographus* (e.g. Christiansen & Bakke, 1988; Grégoire & Evans, 2007). Extensive wind damage or drought increases the risk of intensive spruce bark beetle outbreaks (Marini et al., 2017), as the spruce bark beetles can then attack healthy spruce trees as well. In recent years, insect pests have caused a lot of damage especially in central Europe (Hlásny et al., 2019). Over the last four decades, spruce and pine timber damaged by bark beetles have increased in Europe, from 2.2 million cubic metres of wood (1971–1980) to 14.5 million cubic metres (2002–2010; Seidl et al., 2014). Also, in the southern boreal zone (e.g. in Finland), warm summers have increased the populations of spruce bark beetles, together with unharvested wood left in forests after wind damages (Siitonen & Pouttu, 2014). The insect pest damages are expected to increase in the boreal zone in Northern Europe along with climate warming. In addition, other biotic damaging agents, such as wood decaying *Heterobasidion* root rot, have frequently caused significant economic losses in different areas of Europe (Garbelotto & Gonthier, 2013; Woodward, Stenlid, Karjalainen, & Hüttermann, 1998). Under a warmer climate, a risk of such damage can be expected to increase in coniferous boreal forests as well.

The simultaneous occurrence of multiple hazardous events can make the adverse impacts even manifold. For example, wind and snow‐induced damages may increase the breeding material for bark beetles and increase attacks by *Heterobasidion* species through tree injuries (Honkaniemi, Lehtonen, Väisänen, & Peltola, 2017). Furthermore, the outbreak of bark beetles can increase the amount of easily flammable dead wood exacerbating the forest fire risk (e.g. Jenkins, Runyon, Fettig, Page, & Bentz, 2014). Wood decay also increases the risk of wind damages due to poorer anchorage and stem resistance for trees (Honkaniemi et al., 2017). In the future, the increase of many abiotic and biotic damages in forests may at least partially counteract the positive effects of climate change on forest growth and productivity (Reyer et al., 2017). Accordingly, climate change may cause severe economic losses in European forests (Hanewinkel, Cullmann, Schelhaas, Nabuurs, & Zimmermann, 2013). Similar results have been observed elsewhere in the world. For example, Watt et al. (2019) found that in New Zealand plantation forests, the growth will increase, but also the wind damage and fire risks exacerbate. Also, studies from North America (Gathier et al., 2014; Halofsky et al., 2018; Peterson, Vose, & Patel‐Weynand, 2014) indicate notable climate change risks to forests requiring efficient adaptation measures.

**FIGURE 1:**
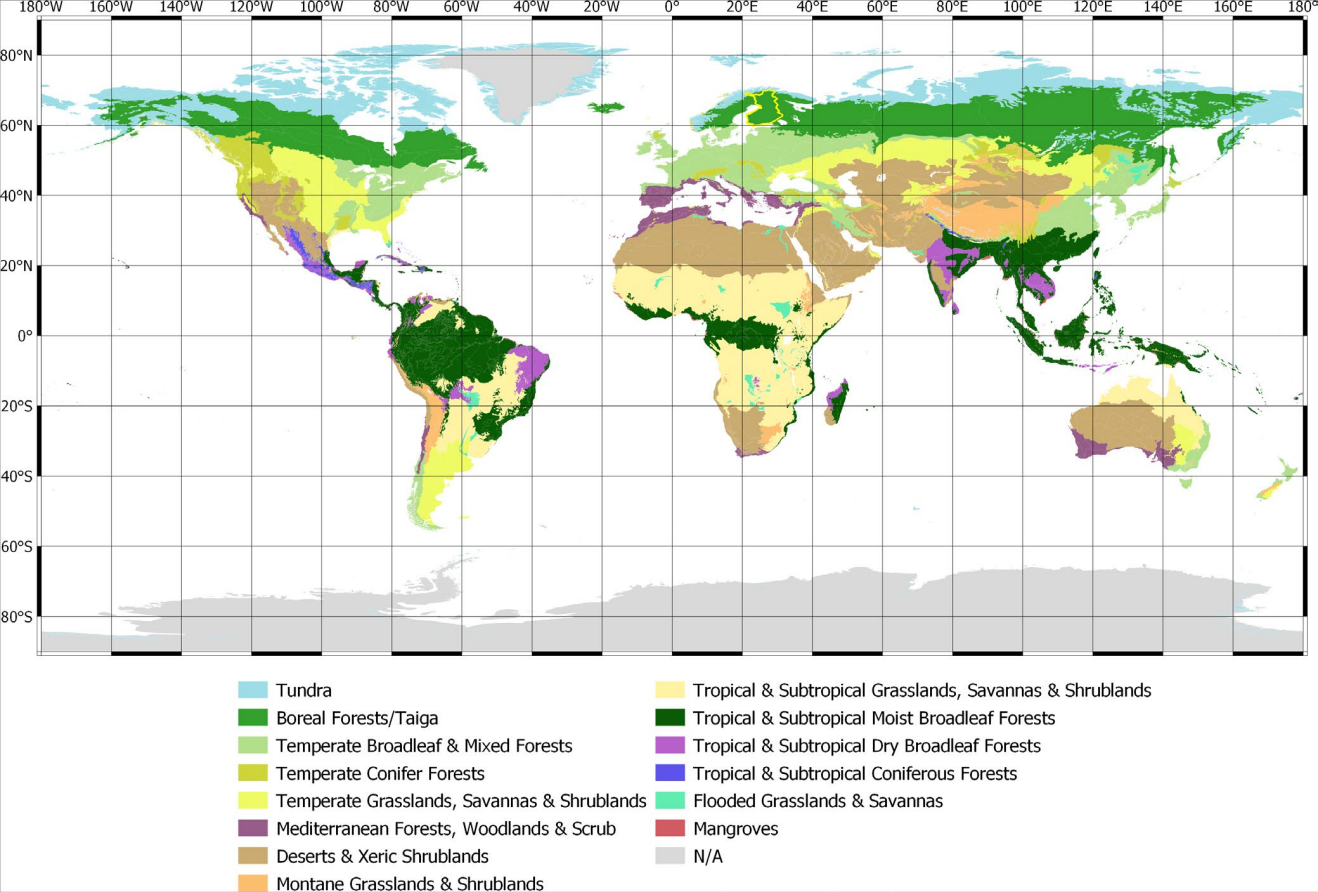
Global biome‐map (Dinerstein et al., 2017). Boundaries of Finland (about 60°N–70°N and 20°E–30°E) are marked in the map

In order to ensure management of forests for different ecosystem services, better preparation is needed in forest management and forestry. This requires a holistic understanding on how the climate change may affect various risks in different spatial and temporal scales. For this purpose, our review article summarizes the most recent findings on major abiotic and biotic risks to boreal forests and forestry under the current and changing climate, with focus on windstorms, heavy snow loading, drought, forest fires and major insect pests and pathogens of trees. Our boreal case study area is Finland, which is an excellent ‘laboratory’ for studying the impacts of climate change in the boreal zone. This is because Finland is the most heavily forested country in Europe and its forests cover about 70% of the land area. Also, the Finnish forests belong to those most intensively monitored in Europe and even globally. Accordingly, even though our review focuses mainly on boreal forests in Finland, the findings evidently have substantial importance also globally and especially in boreal forests, which represent the largest terrestrial biome in the world (Figure 1) and have a significant role in the mitigation of climate change.

## 2 OVERVIEW OF PROJECTED CLIMATE CHANGE IN FINLAND

**FIGURE 2:**
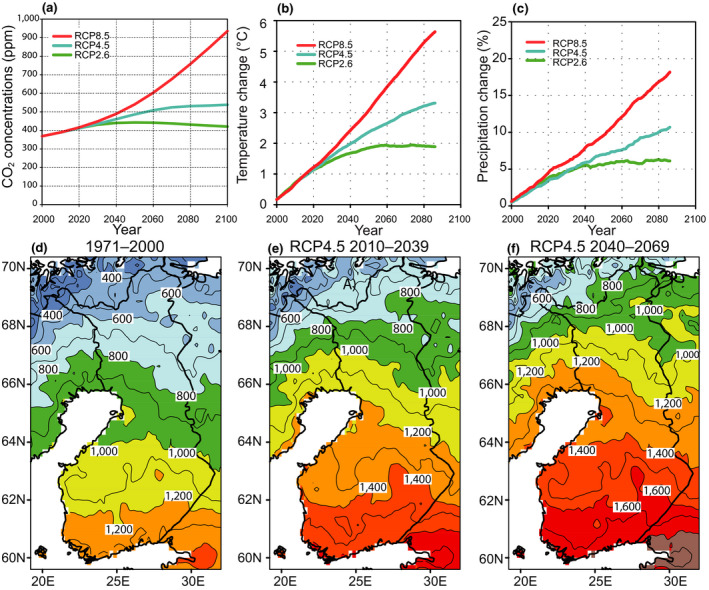
(a) The change of the atmospheric CO_2_ concentration (ppm) in 2000–2100 under three RCP scenarios. (b) The change of the annual mean temperature (°C) and (c) precipitation (%) in Finland in 2000–2085 (relative to the period 1981–2000), both corresponding to the mean of 28 global climate models (Ruosteenoja, Jylhä, et al., 2016). The growing degree days sum (°C day) in (d) 1971–2000, (e) 2010–2039 and (f) 2040–2069 under the RCP4.5 scenario (Ruosteenoja, Räisänen, et al., 2016)

In Finland, the annual mean temperature is now about 2.3°C higher than it was in mid‐19th century (Mikkonen et al., 2015). In the future, according to a multimodel mean, the annual mean temperature is projected to increase by 1.9, 3.3 and 5.6°C by the 2080s under the RCP2.6, RCP4.5 and RCP8.5 scenarios, respectively, compared to the period of 1981–2010 (Figure 2). At the same time, the mean annual precipitation would increase by 6%, 11% and 18% under these RCPs. The changes are projected to be larger during the winter than the summer months. During the potential growing season (April–September), the mean temperature is expected to rise by about 1–5°C and precipitation by 5%–11%, depending on the RCP scenario (Ruosteenoja, Jylhä, et al., 2016).

As a result of the projected warming, higher growing degree day (GDD) sums will accumulate during the longer growing seasons (Figure 2, lower panels). At the end of this century under RCP4.5, the thermal growing season would be 1–1.5 months longer and the GDD sum about 500°C days larger than in 1971–2000 (Ruosteenoja, Räisänen, Venäläinen, & Kämäräinen, 2016).

Compared to other areas of the boreal zone (Figure 1), the summer season temperature increase projected for Finland is of a similar magnitude (IPCC, 2013). In winter, the modelled warming is stronger in northern Russia, Siberia and Alaska than in Finland, particularly in the northern parts of these areas. Moreover, in eastern Siberia and Alaska, summer precipitation is anticipated to increase somewhat more than in Finland, while in southern Canada summers may even become dryer.

## 3 IMPACTS OF CLIMATE CHANGE ON FOREST GROWTH AND TIMBER SUPPLY

**FIGURE 3:**
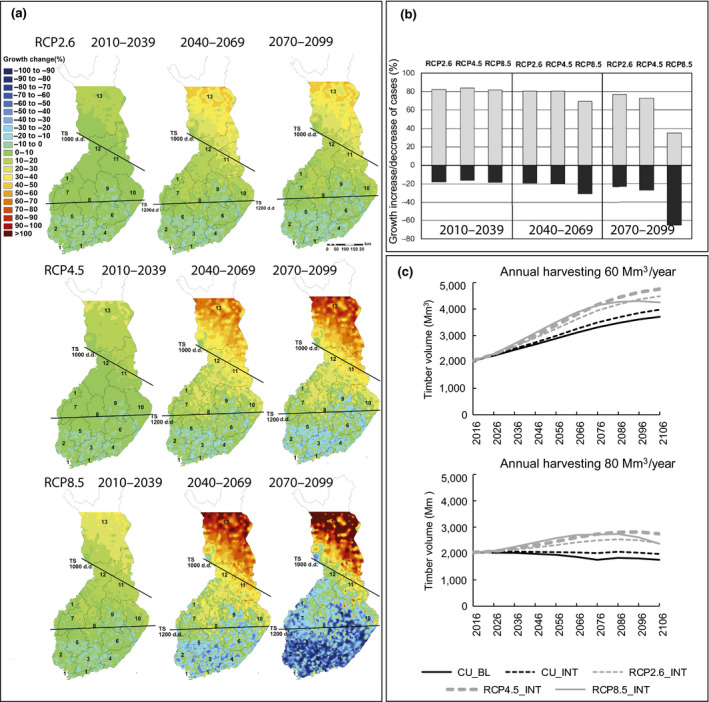
(a) The spatial distribution of the percentage change in diameter growth in Finland over all tree species on upland (mineral) forest inventory plots given separately for three future periods and three RCP scenarios. In the maps the approximate borders of the southern, middle and northern boreal zones (GDD sums 1,000 and 1,200°C days) are shown. (b) The percentage of plots with increasing (grey)/decreasing (black) diameter growth for 2010–2039, 2040–2069 and 2070–2099 under RCP2.6, RCP4.5 and RCP8.5, compared to the period 1981–2010. (c) The total growing stock volume on forest land assigned for timber production in Finland in 2016–2106 with 60 and 80 million m^3^ annual cutting targets of timber under current climate baseline management (CU_BL) and intensified forest management (CU_INT). Also, options of intensified management under climate scenarios are included in the map (RCP2.6_INT, RCP4.5_INT and RCP8.5_INT; Heinonen et al., 2018; Kellomäki et al., 2018)

Based on recent process‐based forest ecosystem model simulations under the RCP2.6, RCP4.5 and RCP8.5 scenarios (Kellomäki et al., 2018), the forest growth evidently increases significantly more in the northern than southern boreal zone, regardless of the RCP scenario (Figure 3a). In the northern boreal zone the growth will also increase clearly more in birch (*Betula* spp.) and Scots pine (*Pinus sylvestris*) than in Norway spruce. On the other hand, when climate change proceeds, in the southern boreal zone, the forest growth may substantially decrease (and mortality increase) under RCP4.5 and especially under RCP8.5, particularly in spruce and to some extent also in pine (RCP8.5), in contrast to birch. The differences in the responses of the tree species and boreal zones increase along with the severity of climate change, which tend to make growing conditions increasingly suboptimal for growth in the south because of too high temperatures and low soil water availability. Forest growth may decline for 20% of the total forest area of Finland under RCP2.6 and RCP4.5 by 2040–2070, and for 30% of the area under RCP8.5 (Figure 3b). Towards the end of the century (2070–2099), the percentage of forest area with declining forest growth increases substantially, being over 60% under RCP8.5.

Moreover, Heinonen et al. (2018) showed that the intensity of forest management and harvesting affects the future forest growth and cutting possibilities (Figure 3c). Under changing climate (especially under RCP2.6 and RCP4.5), with intensified management it is possible to harvest even 80 Mm^3^/year of timber on forest land assigned for timber production during the 90 year period, without decreasing the growing stock volume from the initial one at the country level. They also showed that with intensified management and harvesting of 60 Mm^3^/year, the growing stock volume of forests may be even doubled during the 90 year simulation period, depending on the climate scenario applied. Intensified forest management includes improved regeneration material, forest fertilization and maintenance ditching (in drained peatlands). If drained peatland sites are included in these calculations, the positive growth responses are slightly higher and the negative ones lower (Kellomäki et al., 2018). The possible increase of various abiotic and biotic damage risks to forests and forestry were not considered in the simulations.

## 4 CLIMATE CHANGE‐INDUCED ABIOTIC RISKS TO FORESTS

### 4.1 Wind damage risk to forests

#### 4.1.1 Observed variation of windstorms and strong winds

In Finland, the year‐to‐year fluctuation in the frequency of windstorms is quite high. Southerly and westerly winds are more common than northerly and easterly ones, typically causing wind damage in late autumn when there is no soil frost on ground. However, also in summer months downbursts associated with thunderstorms have sporadically caused large forest damages.

Reliable high‐resolution information on the variations of extreme wind speeds under frozen and unfrozen soil conditions may improve the consideration of wind damage risk in forest management. Laapas, Lehtonen, Venäläinen, and Peltola (2019) estimated the 10 year return levels of the maximum 10 min wind speeds for both frozen (over 20 cm frost depth) and non‐frozen soil periods for dense Norway spruce stands on clay or silt soil and Scots pine stands on sandy soil and on drained peatlands. The coarse‐resolution estimates of the 10 year return levels of the maximum wind speeds derived from the ERA‐Interim reanalysis data (Dee et al., 2011) for 1979–2014 were downscaled to a 20 m grid using the wind multiplier approach, which considers the effects of topography and surface roughness (see e.g. Venäläinen et al., 2017; Yang, Nadimpalli, & Cechet, 2014). The soil frost depth was estimated by using the soil frost model of Lehtonen et al. (2019).

**FIGURE 4:**
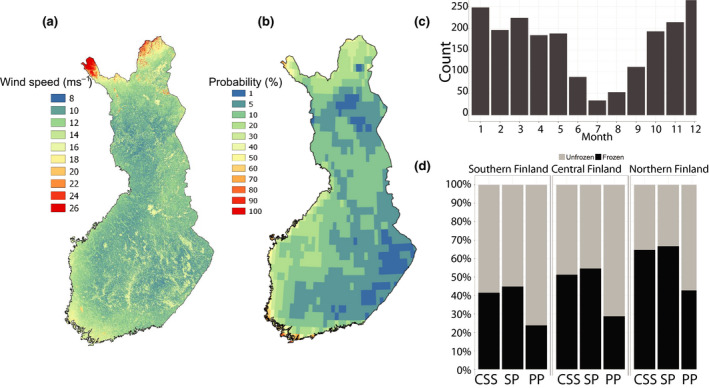
(a) Ten year return level of the maximum wind speed (m/s) derived from downscaled wind data for the years 1979–2014. (b) The annual probability of the gust wind speed exceeding 25 m/s. (c) The monthly frequency of the occurrence of annual maximum wind speed during 1979–2014 at 40 weather stations in Finland. (d) The proportion of the observed annual maximum wind speeds occurring during frozen (black) or unfrozen (grey) soil. The terrain types considered are spruce on clay/silt (CSS), pine on sand (SP) and pine on peat (PP; Laapas et al., 2019)

Laapas et al. (2019) found that in the forested areas of Finland the 10 year return levels of the maximum 10 min wind speed are approximately 12–16 m/s, whereas over the Baltic Sea and large lakes and in the tops of fells in Lapland, the corresponding values are remarkably higher (Figure 4a). The annual maximum wind speeds are typically observed during winter months when strong winds are associated with synoptic scale cyclones (Figure 4c). However, due to the large variability in the timing of annual maximum wind speed, the difference between the frozen and unfrozen season is relatively small (Figure 4d). Overall, the soil frost duration tends to be much shorter in the southern boreal zone, and on peatlands in general, than on upland forest sites in central and northern Finland (Laapas et al., 2019).

Another estimate for the spatial and temporal distribution of devastating winds can be obtained by analysing gust wind speeds that are typically one to two times higher than the 10 min average wind speed. This approach was used by Valta et al. (2019) in the forest wind damage risk assessment. Unfortunately, stations making gust observations have been rare until recent years, and this complicates detailed analysis of the temporal and spatial variation of gusts. However, an estimate can be calculated using the reanalysed meteorological data, like ERA5 with the spatial resolution of about 30 km (C3S, 2017). The reanalysed data represent a value averaged over a grid‐box instead of a point observation, and accordingly the maximum wind speeds are lower compared to point observations. Figure 4b shows the annual probability for the gust wind speeds exceeding 25 m/s that is comparable to a 31–32 m/s wind speed obtained from point observations. Probabilities were derived from hourly data for the years 1979–2018 applying the generalized extreme value distribution of extreme value theory (Coles, 2001) using the R package ‘evd’ (Stephenson, 2002). The annual probability of the 25 m/s exceedance varies from approximately 60% on the Baltic Sea coast to values close to 1% in eastern Finland.

According to Laapas and Venäläinen (2017), in Finland, the occurrence of strong winds has been decreasing during the recent years. During the period 1959–2015, the mean linear trends for the annual mean and maximum wind speeds were −0.09 and −0.32 m/s per decade respectively. This observed weakening of winds in Finland agrees with the global stilling of terrestrial near‐surface wind speeds (McVicar et al., 2012). Possible reasons for stilling may be the increased surface roughness related to the increased volume of growing stock of forests and changes in atmospheric circulation (Vautard, Cattiaux, Yiou, Thépaut, & Ciais, 2010).

#### 4.1.2 Projected future changes in windstorms and strong winds

**FIGURE 5:**
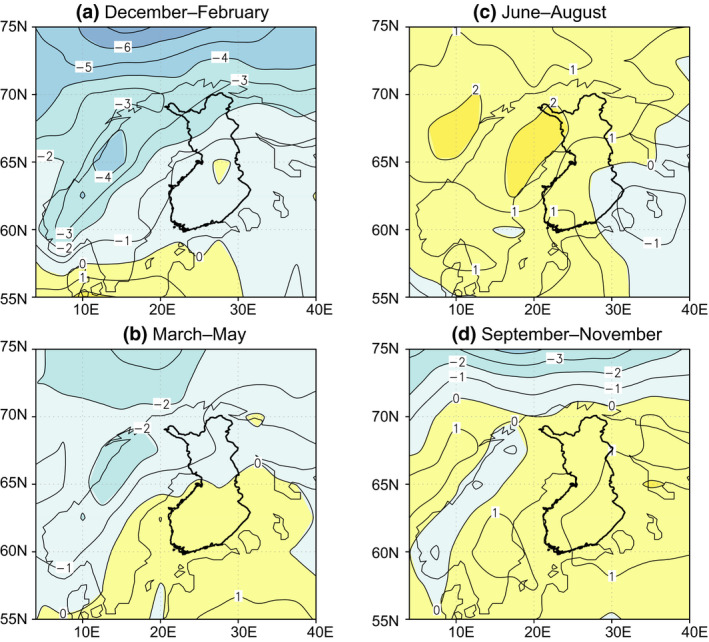
Projected multimodel mean change (in %) in the 99th percentile of the geostrophic wind speed between 1961–2005 and 2040–2069 under RCP8.5 in (a) December–February, (b) March–May, (c) June–August and (d) September–November (Ruosteenoja et al., 2019)

Recent simulation studies (Groenemeijer et al., 2016; Kjellström et al., 2018; Ruosteenoja, Vihma, & Venäläinen, 2019), do not indicate any robust climate change signal in the future occurrence of strong winds in Nordic countries. Ruosteenoja et al. (2019) derived the projected changes in geostrophic winds from 21 global climate models (GCMs). These estimates can be regarded as more reliable than the projected changes in near surface winds, which are very dependent on the surface characteristics specified in the climate model. In Figure 5 an estimate for the change of the 99th percentile of the frequency distribution of geostrophic winds is given, that is, for the highest 1% of wind speeds. According to this estimate, strong winds may increase slightly, by 0%–2%, in summer and autumn. In spring and winter, the projected change is even more negligible. Moreover, these projected changes are subject to significant intermodel scatter. In addition, the proportion of westerly winds can increase slightly at the cost of easterly winds.

Soil frost that is regarded as beneficial for forestry as it anchors trees to the ground is predicted to decrease, both in depth and duration (Kellomäki et al., 2010; Lehtonen et al., 2019; Venäläinen et al., 2001). In southern and central Finland, and likewise on peatlands in large parts of Finland, later in this century there would be only short periods of deeply frozen soil in most of winters (Lehtonen et al., 2019). Therefore, wind‐ and snow‐induced damages may be expected to increase in the future, especially in southern and central Finland.

#### 4.1.3 Magnitude of observed and predicted wind damage in forests

In Finland, over 24 million m^3^ of timber in total has been damaged in different winter and summer storms since 2000 (e.g. Kärhä et al., 2018; Zubizarreta‐Gerendiain, Pukkala, & Peltola, 2017). Typically, during unfrozen soil conditions the occurrence of wind damage has required 10 min mean wind speeds of about 13–18 m/s (in gusts up to 30 m/s). On the other hand, late autumn and winter storms accompanied with heavy snowfall have caused damage even with a wind speed of 8–13 m/s, as was observed in the Pyry storm in November 2001 (e.g. Zubizarreta‐Gerendiain, Pellikka, Garcia‐Gonzalo, Ikonen, & Peltola, 2012). The most severe individual windstorms have typically damaged 2–4 million m^3^ of timber. Also, convective summer events can result in the same scale of damage; for example, in 2010 four thunderstorms together damaged over 8.1 million m^3^ of timber (Viiri et al., 2011).

When comparing the volume of observed wind damage to the maximum observed inland wind gust speeds during the same severe windstorms (convective summer events excluded), Valta et al. (2019) found that the volume of damage is approximately proportional to the 10th power of wind gust speed. If the maximum wind gust speed exceeds about 30 m/s, forest damage exceeds million m^3^.

**FIGURE 6:**
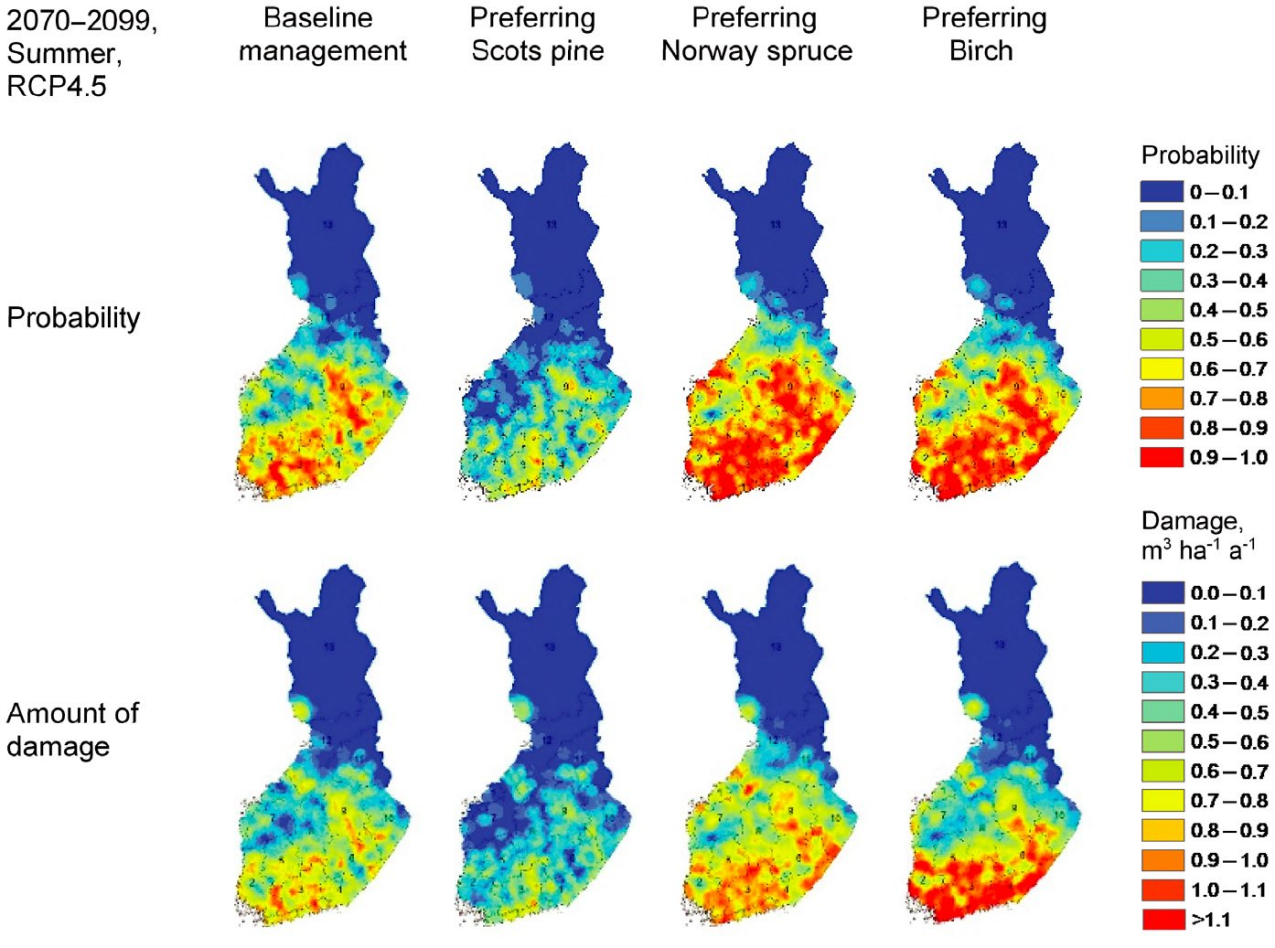
Probabilities from 0 to 1 of wind speeds causing damage, and the estimated amount of damage (m^3^/ha) in summer (birch in leaf) on upland forest inventory plots throughout Finland under the RCP4.5 scenario during 2070–2099 for different management regimes (based on data from Ikonen et al., 2017)

Ikonen et al. (2017) simulated potential future wind damages with a forest ecosystem model (SIMA) and a mechanistic wind damage risk model (HWIND). The results indicate that the probability and the predicted amount of wind damage are in general clearly higher in the southern boreal zone with a dominance of Norway spruce (and birch) stands than in the northern boreal zone with a dominance of Scots pine (Figure 6). Furthermore, they showed that preferring Norway spruce or birch in tree planting clearly increases the probability for wind damage compared to preferring either Scots pine or the business as usual (baseline) management, in which the same tree species is planted after the clear‐cut. This is because much lower wind speed is required to damage Norway spruce compared with Scots pine or birch of same tree size.

### 4.2 Snow damage

After windstorms, heavy snow loading is nowadays the most important abiotic cause of damage in the Finnish forests. Snow‐induced forest damages include stem breakage and bending or leaning of stems. If the soil consists of unfrozen trees they can also be uprooted (e.g. Nykänen et al., 1997). In Finland, snow damage has been detected at 7.1% of forested land, less extensively in the south (3.4%) than north (12.1%; Korhonen et al., 2017). Snow‐induced damage has typically occurred when the load of wet snow on tree crowns has exceeded 30 kg/m^2^ (Nykänen et al., 1997). Young Scots pine and broadleaf stands, especially those with a high height to stem diameter ratio, are particularly susceptible to snow damage.

**FIGURE 7:**
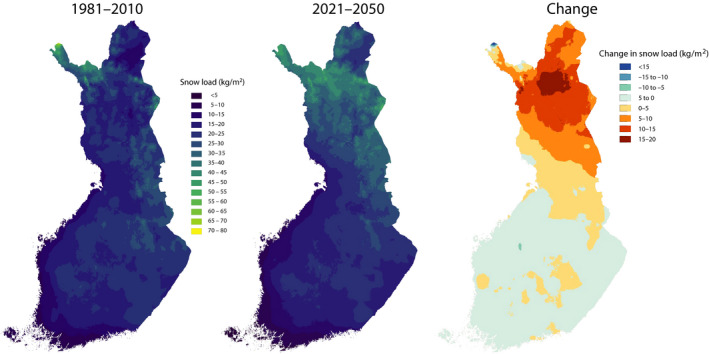
Annual maximum snow load on tree crowns in 1981–2010 (left), 2021–2050 (middle) and the change from 1981–2010 to 2021–2050 (right) under RCP4.5 (based on Lehtonen, Kämäräinen, et al., 2016)

Lehtonen, Kämäräinen, Gregow, Venäläinen, and Peltola (2016) estimated the impacts of climate change on snow loads on tree crowns by applying a snow load model developed at the Finnish Meteorological Institute (Lehtonen, Hoppula, Pirinen, & Gregow, 2014). The model simulates the accumulation of snow load on tree crowns including rime, frozen, wet and dry snow loads. Their results indicate increasingly heavy snow loads and risks for snow damage in eastern and northern Finland, opposite to southern and western Finland (Figure 7). The projected change is rather similar both for heavy rime, wet snow and frozen snow loads. Conversely, dry snow loads were predicted to decrease nearly everywhere in Finland. The predicted increase in the risk of snow damages in eastern and northern Finland is induced by the increasing frequency of wet snow hazards and conditions favourable for rime accumulation in the increasingly humid and mild but still cold enough climate.

### 4.3 Drought and forest fires

Climate warming is expected to increase the occurrence of drought in northern Europe, despite the simultaneous increase in precipitation, because higher temperatures act to increase potential evaporation, which will overcome the impact of increased precipitation. In spring and early summer especially, the average moisture in the soil surface layer will decrease under the climate warming (Ruosteenoja, Markkanen, Venäläinen, Räisänen, & Peltola, 2018). Also, the probability of anomalously dry events will increase.

Climate change likewise acts to increase the risk of large‐scale forest fires in Finland (Lehtonen, Venäläinen, Kämäräinen, Peltola, & Gregow, 2016; Venäläinen, Lehtonen, & Mäkelä, 2016). The occurrence of a forest fire requires favourable weather conditions, such as strong winds, high temperatures and low air humidity. Until today the Finnish forest fire season has typically been quite short, extending from May to September. Venäläinen et al. (2016) have applied forest fire indexes like the Canadian Fire Weather Index (Van Wagner, 1987) and the Finnish Forest Fire risk index (Vajda, Venäläinen, Suomi, Junila, & Mäkelä, 2014) to estimate the frequency of the most severe risk conditions for large fires in Finland. They reported a high variability in the fire danger. For example, in 1999, 2002 and 2006, high fire danger conditions in southern Finland lasted almost the whole summer, whereas in the rainy summer of 1998 there were only a few days with high fire danger. In northern Finland, the fire season is shorter and the number of days with fire danger in general was lower. Venäläinen et al. (2016) estimated that the fire risk is high simultaneously throughout Finland approximately once in a decade, as a result of the occurrence of widespread drought together with high temperatures, low air humidity and high wind speed. Such a high fire risk (in terms of fire‐danger index values) is typical mainly in southern Europe and the Mediterranean countries. On the other hand, individual days exist when the spread of fires into conflagrations is possible over some smaller area almost every year in southern Finland but more rarely in the north.

**FIGURE 8:**
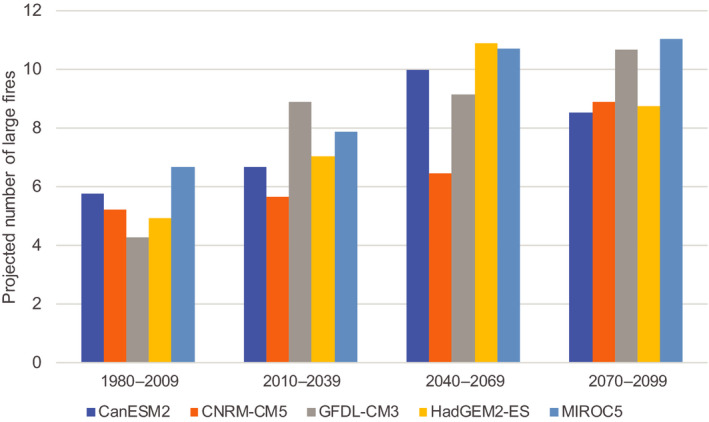
The impact of climate change on the number of potential forest fires larger than 10 hectares under the RCP4.5 scenario, compared with the period 1980–2009. Bars indicate the estimates based on different climate models used in the study (Lehtonen, Venäläinen, et al., 2016)

Lehtonen, Venäläinen, et al. (2016) studied the impacts of climate change on the risk of large forest fires using the seasonal severity rating index included in the Canadian Fire Weather index system (van Wagner, 1987). Even though the annual precipitation total in Finland is projected to increase, the sign of change in the summer (forest fire) season is somewhat uncertain (Ruosteenoja, Jylhä, et al., 2016). Lehtonen, Venäläinen, et al. (2016) showed that under the RCP4.5 scenario, the number of large (>10 ha) forest fires may increase by several tens of percentage in the periods of 2010–2039, 2040–2069 and 2070–2100, respectively, compared with the period of 1980–2009 (Figure 8). When estimating the risk of large forest fires, inclusion of other influencing factors, in addition to weather, would make analyses more robust. For example, the effectiveness of fire detection and fire suppression determines how widely fires spread. In Finland, making open fires under high‐risk conditions is forbidden and the ban is generally well respected. If the attitude of people on fire risk warnings and handling of fire in risky conditions becomes more neglectful and it will affect the occurrence of fires adversely.

## 5 CLIMATE CHANGE‐INDUCED BIOTIC RISKS TO FORESTS

### 5.1 Insect pest damages

#### 5.1.1 Spruce bark beetle outbreaks

Nowadays the most devastating insect pest in Finnish forests is the European (spruce) bark beetle (*I. typographus*; Asikainen et al., 2019), the reproduction of which will benefit on higher summer temperatures. Spruce bark beetles can generate two generations in one summer if the GDD sum exceeds approximately 1,500°C days, that is, 2× the GDD sum needed for the complete development of an individual from the egg to adult (625–750 GDD; Jönsson, Harding, Bärring, & Ravn, 2007). Moreover, the number of successfully developed beetles in different sister broods of the first generation increases with the increase of the GDD sum (Öhrn, Långström, Lindelöw, & Björklund, 2014).

Traditionally, spruce bark beetle has been a univoltine species in Finland and existed in low numbers and mainly on windthrown trees (Annila & Petäistö, 1978). However, in recent years, it has become more abundant. In the early 2000s, there was a regional outbreak in southern Finland (Eriksson, Pouttu, & Roininen, 2005). As a result of the exceptionally warm thermal growing season in 2010, the number of successfully developed individuals in the first generation of the season was high, and spruce bark beetles were able to produce a second generation during the same year (Öhrn et al., 2014; Pouttu & Annila, 2010). In the summer of 2010, drought stress also weakened the defence of spruces against the insect pests (Siitonen & Pouttu, 2014). Additionally, there was a lot of breeding material in forests as a result of strong thunderstorms, which damaged a total of 8.1 million m^3^ of timber between late July and early August 2010 (Viiri et al., 2011). The next summer 2011 was likewise warm, and a severe windstorm in December 2011 caused additional extensive damage in the forests. Therefore, conditions for the growth of spruce bark beetle populations were favourable in 2012 as well. The population peak was achieved in summer 2013, when beetle damage was widely observed in living trees in south‐eastern Finland.

Overall, in the 20th century, the annual GDD sum in southern Finland mainly varied between 1,200 and 1,400°C days and only occasionally exceeded the threshold of 1,500°C days. During the early 21st century, by contrast, the GDD sums have tended to exceed this threshold frequently. Between 2000 and 2019, high GDD sums have created conditions suitable for the occurrence of a second generation of spruce bark beetle in southern Finland (Kniivilä et al., 2020). The summer of 2018 was record warm, and the highest GDD sums in southern Finland reached 1,900°C days and even in central Finland GDD exceeded 1,500°C days.

**FIGURE 9:**
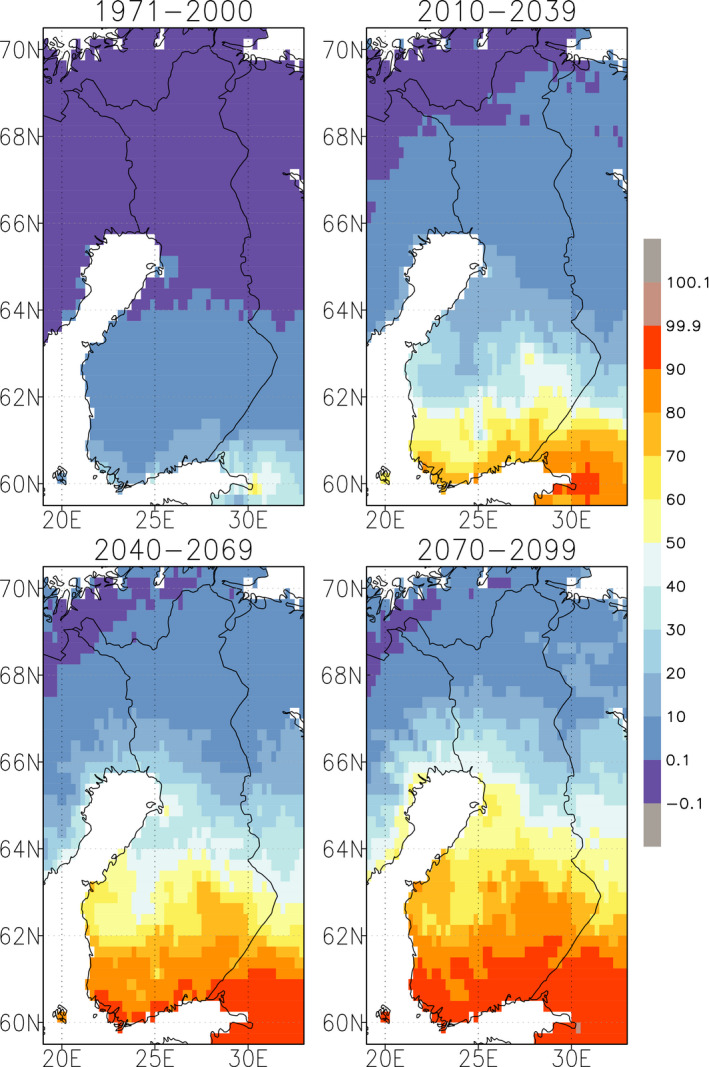
The probability of the growing degree day sum exceeding 1,500°C days for the periods 1971–2000, 2010–2039, 2040–2069 and 2070–2099 under RCP4.5, derived from simulations performed with 23 global climate models (Ruosteenoja, Räisänen, et al., 2016)

Asikainen et al. (2019) explored the probability of exceeding the GDD sum of 1,500°C day threshold in Finland for the period between 1971 and 2000, and for the future periods of 2040–2069 and 2070–2099 under the RCP4.5 scenario, based on the previous work by Ruosteenoja, Räisänen, et al. (2016). In 1971–2000, the probability of the GDD sum exceeding 1,500°C days was below 10% in southern and central Finland (apart from the southern coast) and below 1% in northern Finland (Figure 9). In 2040–2069, the probability of GDD sums of 1,500 or higher will already be >80% in southern Finland, about 60% in the central parts and 10%–30% in southern Lapland. In 2070–2099, the probability of such a high GDD sum is >90% in southern, 70% in central and 10%–40% in southern Lapland (Figure 9, bottom right panel). The findings of Asikainen et al. (2019) are in line with the recent study of Bentz et al. (2019) who used regionally downscaled GCM data together with the phenology model for development of spruce bark beetle (including the onset of swarming, development from an egg to an adult and the onset of reproductive diapause), developed by Jönsson et al. (2011).

#### 5.1.2 Other insect pests

Large pine weevil (*Hylobius abietis*) is a major problem for the regeneration of coniferous forests in Europe (Långström & Day, 2004). In Finland, it is the only forest insect pest which needs continuous active measures in order to minimize its damages on planted tree seedlings. The damage caused by the large pine weevil is expected to increase in the future. Warmer summers and shortening of the period when soil is frozen shortens the development time of immature weevils, increases feeding and prolongs feeding period. Historically, the development time of the large pine weevils has been 2 years in southern and more than 3 years in northern Finland, and the abundance of weevils in clear‐cuts decreases from south to north (Långström, 1982). In a warmer climate, a new generation of weevils will emerge sooner, at the time when planted seedlings are still small and vulnerable to serious feeding damage. The study for northern Sweden also suggested that warming of climate will increase the weevil damage risk (Nordlander, Mason, Hjelm, Nordenhem, & Hellqvist, 2017).

Warming of climate is anticipated to benefit several other insects which have frequently caused significant defoliation on trees, because many outbreaking insect defoliators overwinter as eggs exposed to extreme low temperatures. So far, frequent cold winters have limited their populations, but an increase of the winter temperatures will favour their reproduction. A well‐known example is the outbreak of autumnal moth (*Epirrita autumnata*) and winter moth (*Operophtera brumata*) in mountain birch forests of northern Fennoscandia; these moths have spread to new areas because of the increase of winter temperatures (Jepsen, Hagen, Ims, & Yoccoz, 2008). In addition, the populations of the pine sawfly (*Neodiprion sertifer*), which has been historically the most significant defoliator of pines in Finland (Juutinen & Varama, 1986), may increase in eastern and northern Finland due to the rising winter temperatures and in southern and western Finland due to the increasing summer drought (Nevalainen, Sirkiä, Peltoniemi, & Neuvonen, 2015; Virtanen, Neuvonen, Nikula, Varama, & Niemelä, 1996).

In addition to insect defoliators overwintering as an egg, some defoliating species overwinter as a pupa. This group includes several species which have caused only occasional severe outbreaks in Finland, but which frequently cause severe defoliation in central Europe. If the future temperature sums in Finland will be close to those currently recorded in central Europe, such species will have a high risk to cause damage in Finland. These species include Pine beauty moth (*Panolis flammea*), pine lopper moth (*Bupalus piniarius*) and common pine saw‐fly (*Diprion pini*; Sierota, Grodzki, & Szczepkowski, 2019).

### 5.2 Forest pathogens

#### 5.2.1 *Heterobasidion* species

The most devastating forest pathogen in Finland currently is *Heterobasidion* spp, which is the most important wood‐decay fungi for conifers in Europe and even globally (Woodward et al., 1998). In Finland, two species of *Heterobasidion* occur namely, *H. annosum* and *H. parviporum*. *H. annosum* is economically the most important forest pathogen in northern Europe, causing root and butt rot diseases on pine trees. It is most common in south‐eastern parts of the country. In Finland, *H. parviporum* is a more common species than *H. annosum*, and it causes root and butt rot on spruces. In southern Finland, approximately 15%–20% of spruces suffer from the root rot. *H. parviporum* occurs sporadically also in northern Finland, although root and butt rot diseases of conifers are caused mainly by other decay fungi. (Müller, Henttonen, et al., 2018; Müller, Kaitera, & Henttonen, 2018). Increasing temperatures increase the spore formation of the *H. parviporum*, and it is expected that the share of infected spruces will increase in the future (Müller et al., 2015; Pukkala, Möykkynen, Thor, Rönnberg, & Stenlid, 2005). In an infected stand, the vegetative spread of the fungus can be very effective. The higher temperatures will enhance the growth rate of *H. parviporum*, since the optimal temperature for the growth mycelia is between 20°C and 30°C (Müller et al., 2015). Higher growth rates of mycelia act to increase the amount of decay in infected trees and the spread of fungus in diseased stand. Furthermore, the decrease of soil frost duration under the warming climate may increase root damages in forest harvesting, making trees more vulnerable for wood‐decay fungus.

#### 5.2.2 Other forest pathogens

Very little is known about the effect of climate change on other major forest pathogens in Finland. An ascomycete fungus *Gremmeniella abietina* has caused major epidemics in pine forest in Finland and Sweden (Nevalainen et al., 2015; Wang, Stenström, Boberg, Ols, & Drobyshev, 2017). The infection of plant tissues by airborne spores of fungi is largely dependent on air moisture at the time of spore dispersal. It is known that successive years with moist and cool springs are followed by major epidemics of *G. abietina*. We can assume that an increase of spring temperatures and drought periods will limit the epidemics of *G. abietina* and other pathogens which infect the shoots and foliage of trees.

### 5.3 Mammalian herbivores

The browsing caused by local high moose (*Alces alces*) populations is one of the most serious forest damage problems in Finland in Scots pine and birch forests. This has led to an increase of cultivation of Norway spruce on less fertile forest sites that are more suitable for pine. The impact of climate change on the risks of moose‐caused forest damages is more difficult to evaluate than the impacts of insect and fungal forest pests. The reduction of the snow depth and duration under climate change may increase the severity of browsing damage (Herfindal, Tremblay, Hester, Lande, & Wam, 2015). It is also possible that in southern Finland dense moose populations will be partially replaced by an increasing deer population (Weiskopf, Ledee, & Thompson, 2019). Indeed, since 2010, the white tail deer (*Oedocoileus virginianus*) population has increased exponentially, thus already being more numerous than moose in southwestern Finland. The effect of an increasing deer population on forest damage in Finland is unknown. Elsewhere, such dense populations have led to overbrowsing and severe problems in the regeneration of deciduous trees and other preferred tree species (Côté, Rooney, Tremblay, Dussault, & Waller, 2004; White, 2012).

In addition to cervids, the length of snow cover will affect small mammals. In a warming climate, there can be changes or even dampening in the vole cycles (Cornulier et al., 2013). Climate warming may also increase the abundance of herbivores, including voles. However, the impact of warming on future vole damage in Finland is still largely unknown.

### 5.4 New biotic threats to forest health

In addition to old and familiar problems, new issues will almost certainly emerge as the climate warming proceeds. An example of the potential new threats is the recent northward spread of nun moth, *Lymantria monacha*. It is nowadays capable of causing large‐scale defoliation in coniferous forests in central Europe (Bejer, 1988; Nakládal & Brinkeová, 2015; Sierota et al., 2019). Nun moth has been extremely rare in Finland (Saalas, 1949), but because of warming of winters, nun moth populations have spread northwards (Fält‐Nardmann et al., 2018). Melin, Viiri, Tikkanen, Elfving, and Neuvonen (2020) likewise reported that this species is now common throughout southern Finland and can be locally highly abundant.

New, invasive alien species can also be accidentally introduced by international plant trade (Hantula, Müller, & Uusivuori, 2014; Lilja et al., 2011). Cold climate has so far protected Finnish forests from alien species, and their numbers are low. However, there are some examples indicating the potential threat to forest health caused by invasive alien species. Ascomycete fungus *Hymenoscyphus pseudoalbidus*, causing ash dieback, has already spread to ash trees in southern Finland. Another example is the longhorn beetle, *Anoplophora glabripennis*, which was found but successfully eradicated from one location in Southern Finland. There are several unfortunate examples of forest health hazards caused by alien invasive species around the world, and globally they are regarded as one of the most significant threats to natural and planted forest ecosystems (Aukema et al., 2011; Pautasso, Schlegel, & Holdenrieder, 2015; Wingfield, Brockerhoff, Wingfield, & Slippers, 2015).

## 6 DISCUSSION AND CONCLUSIONS

**FIGURE 10:**
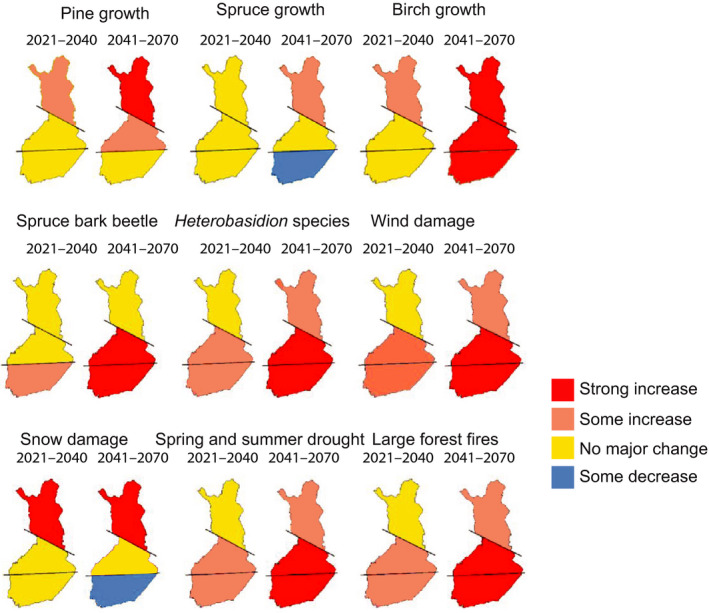
Estimated impacts of climate change (corresponding to RCP4.5) on forest growth and the major climate‐induced risks (edited from Äijälä, Koistinen, Sved, Vanhatalo, & Väisänen, 2019). The baseline period is 1981–2010

Warmer and drier summer conditions are expected to increase droughts, forest fires and pest insects, while warmer and wetter winters will lead to increasing damage by windstorms, heavy snow loading and pathogens (Jactel et al., 2011; Reyer et al., 2017; Seidl et al., 2017). Such disturbances are likely to be the most pronounced in coniferous forests, particularly in the boreal zone (Seidl et al., 2017). Therefore, to ensure preconditions for sustainable multifunctional forest management and use of different ecosystem services, a holistic understanding is required on how climate change may affect abiotic and biotic risks in different spatial and temporal scales. For this purpose, our review article summarized the most recent findings on the major abiotic and biotic risks to boreal forests and forestry in Finland under the current and changing climate (see Figure 10).

Depending on the development of the GHG concentrations, the mean annual temperature in Finland is projected to increase in the range of 1.5–6°C by 2100, compared to the period 1981–2000 (Ruosteenoja, Jylhä, et al., 2016). In general, the forest growth and productivity tend to increase, mainly in the northern boreal zone (Kellomäki et al., 2018). Conversely, in the southern boreal zone, a large increase in summer temperatures and associated drought (especially under RCP4.5 and RCP8.5) may make growing conditions suboptimal, especially for Norway spruce but partially for Scots pine as well (Kellomäki et al., 2018). Accordingly, in this zone there may be a need to avoid the cultivation of spruce, particularly on forest sites with a relatively low water holding capacity (Figure 10).

In Finland, in recent decades there has occurred less wind damage than elsewhere in Europe, including Sweden. However, the wind damage risk of forests is expected to increase along with climate warming, especially in the southern and middle boreal zones of Finland, mostly in stands dominated by Norway spruce with shallow rooting (Ikonen et al., 2017). This is so despite any significant change in wind climate, because of shortening of the soil frost period, particularly in the southern and middle boreal zones and on peatlands in large parts of Finland (Lehtonen et al., 2019). As a result, strong winds in the future will blow more frequently under unfrozen soil conditions (Kellomäki et al., 2010; Laapas et al., 2019; Peltola, Kellomäki, & Väisänen, 1999). The increasing risks of wind damage should therefore be considered in forest management, for example, in planning of temporal and spatial patterns of thinnings and clear‐cuts (Heinonen et al., 2011). Moreover, the increasing risk of snow damage, especially in Scots pine and birch stands in the eastern and northern Finland should be considered in forest management, for example, by making timely precommercial and commercial thinnings and avoiding forest fertilization on forest sites located more than 200 m above the sea‐level (Nykänen et al., 1997; Valinger & Lunqvist, 1992).

The increase in summer droughts (Ruosteenoja et al., 2018) may also exacerbate the risk of large‐scale forest fires, especially in southern and middle boreal conditions (Lehtonen, Venäläinen, et al., 2016; Mäkelä, Venäläinen, Jylhä, Lehtonen, & Gregow, 2014) which should be considered in forest harvesting operations. In southern and middle boreal zone especially, the warmer climate may make coniferous forests more liable to bark beetle outbreaks and to the wood‐decaying *Heterobasidion* species (Asikainen et al., 2019). Furthermore, that kind of devastating biotic damages that have already been observed in central Europe (Hlásny et al., 2019; Schelhaas et al., 2003; Seidl et al., 2014; Woodward et al., 1998), may become more common in the future, especially in coniferous forests in the southern and middle boreal zone of Finland. In this respect, multifunctionality of forest management (Díaz‐Yáñez, Pukkala, Packalén, & Peltola, 2019; Seidl & Lexer, 2013) is needed to increase the resilience of forests to multiple disturbances induced by the climate change (Seidl et al., 2017). For example, growing mixed forests on suitable forest sites instead of monocultures may increase the resilience.

**FIGURE 11:**
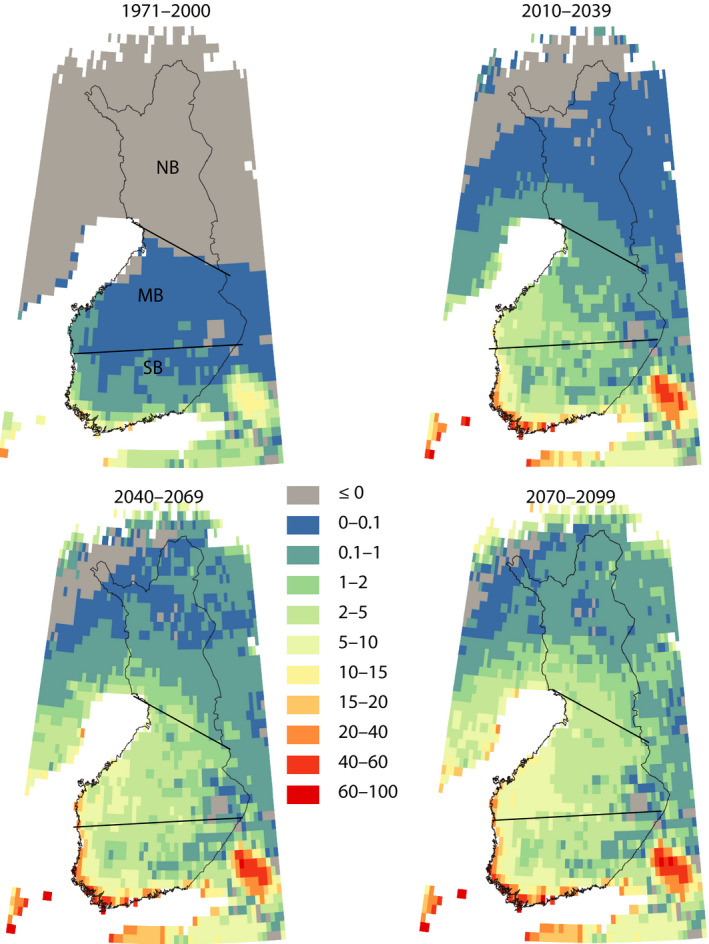
The annual probability for climatological conditions optimal for an outbreak of a serious bark beetle invasion. The probability is calculated by multiplying the probability of a large wind damage (Figure 4) by the probability of growing degree day exceeding 1,500°C day (Figure 9). Approximate borders of the southern (SB), middle (MB) and northern (NB) boreal zones are drawn in the figures

The simultaneous consideration of multiple risks of forests should also be emphasized in the future forest management and forestry. This is important, in particular, because the frequency of harmful cascading events is likely to increase. One example of such an event chain is a large‐scale wind damage producing substantial amount of unharvested damaged wood in the forests, followed by a very warm summer favouring bark beetle invasion (Lindelöw & Schroeder, 2008). Such a cascading damage chain can lead to far larger losses compared with a situation when these events happen independently. For example, if the storm takes place in December, the windiest month of the year (Laapas et al., 2019), there is roughly a 6‐month period to harvest all the damaged wood prior to the beginning of the spruce bark beetle breeding period. If the damage occurs during spring, the period available to collect the damaged wood becomes far shorter. A windstorm characterized by measured wind gust at a speed of 31–32 m/s (equalling approximately the 25 m/s in the ERA5 reanalyses) would initiate wind damage exceeding 2–3 million m^3^. This is close to the upper limit of the timber amount that can be collected from the forests with the existing timber harvesting resources (Valta et al., 2019). The total probability for conditions optimal for large bark beetle damages is thus the probability of a windstorm causing large forest damages (Figure 4) multiplied by the probability of the GDD sum exceeding 1,500°C days during the next summer (Figure 9). In recent past climate (1971–2000), the risk was approximately 2%–5% on the south‐western coast of Finland, less than 1% in the central parts of Finland and in the north virtually zero (Figure 11). At the end of this century, in the southern boreal zone, the annual probability will be about 40% at the coast of the Baltic Sea and about 2%–5% in the eastern part of the zone. In the western parts of the middle boreal zone, the probability is about 5%–20%, decreasing to about 1%–5% in the east. In the northern boreal zone, the probability ranges from about 5% in the southern parts to about to less than 1% in the northern parts of the zone and the country (Figure 11). Accordingly, compared with 1971–2000, climate warming will drastically increase the occurrence of conditions favourable for a widespread spruce bark beetle outbreak.

The above narrative demonstrates illustratively how seriously climate change can influence boreal forests in the future. The next step in the damage chain would be an ignition of large forest fires, as has happened in Canada. A robust assumption is that climate change will also manifold the number of forest fires and increase burned area, as shown by Lehtonen, Venäläinen, et al. (2016). Recent mega‐scale forest fires in Canada and Siberia have also released huge amounts of stored carbon into the atmosphere, thus nullifying the potential positive impact of climate change on carbon sequestration in forests (Walker et al., 2019).

According to our study gradual climate change generally tends to accelerate forest growth, however, drought and biotic damages in particular may have adverse impacts. When comparing conditions in Finland with those in North America, the largest difference lies in the magnitude of disastrous events, like forest fires and insect‐caused damages. Until today, in Finland the control of forest fires has succeeded well, and the burned area has been only a few hundred hectares annually (Lehtonen, Venäläinen, et al., 2016), while in Canada the annual burned area is typically millions of hectares (Hanes et al., 2019). When climate change proceeds, the environmental conditions are foreseen to become more severe in Finland. This likewise holds for the biotic damage risks as demonstrated in Figure 11; the risks have already been observed to increase in Central Europe (see Seidl et al., 2014).

Luyssaert et al. (2018) have emphasized that the primary role of forest management in Europe in the coming decades is not to protect the climate, but to adapt the forest cover to changing climatic conditions. In the future, the probability of harmful multiple and cascading forest disturbance events may increase remarkably in boreal conditions. Therefore, the simultaneous consideration of multiple risks of forests should be emphasized in forest management and forestry. This is also needed in order to ensure preconditions for multifunctional forest management and simultaneous provisioning of different ecosystem services in a sustainable way. The climate change‐induced risks can to some extent be mitigated through forest management practises, like making forest thinning and cutting the way that the wind, snow and fire risks are minimized. One potential, but little studied option is to grow trees in mixed species stands as an attempt to lower the risk of biotic forest health hazards (Jactel & Brockerhoff, 2007).

## Data Availability

The data that support the new finding (Figure 11) of this study are available from the corresponding author upon reasonable request.
